# Comparing interferon-gamma release assays with tuberculin skin test for identifying latent tuberculosis infection that progresses to active tuberculosis: systematic review and meta-analysis

**DOI:** 10.1186/s12879-017-2301-4

**Published:** 2017-03-09

**Authors:** Peter Auguste, Alexander Tsertsvadze, Joshua Pink, Rachel Court, Noel McCarthy, Paul Sutcliffe, Aileen Clarke

**Affiliations:** 1 Warwick Evidence, Warwick Medical School, University of Warwick, Coventry, CV4 7AL UK; 2 Evidence in Communicable Disease Epidemiology and Control, Health Sciences, Warwick Medical School, University of Warwick, Coventry, UK

**Keywords:** Systematic review, Latent tuberculosis infection, Interferon gamma release assays, Tuberculin skin test

## Abstract

**Background:**

Timely and accurate identification of people with latent tuberculosis infection (LTBI) is important for controlling *Mycobacterium tuberculosis* (TB). There is no gold standard for diagnosis of LTBI. Screening tests such as interferon gamma release assays (IGRAs) and tuberculin skin test (TST) provide indirect and imperfect information. This systematic review compared two types of IGRAs QuantiFERON®-TB Gold In-Tube test (QFT-GIT) and T-SPOT.TB with TST for identification of LTBI by predicting progression to a diagnosis of active TB in three subgroups: children, immunocompromised people, and those recently arrived from countries with high TB burden.

**Methods:**

Cohort studies were eligible for inclusion. We searched MEDLINE, EMBASE, the Cochrane Library and other databases from December 2009 to June 2015. One reviewer screened studies, extracted data, and assessed risk of bias with cross checking by a second reviewer. Strength of association between test results and incidence of TB was summarised using cumulative incidence ratios (CIRs with 95% CIs). Summary effect measures: the ratio of CIRs (R-CIR) with 95% CIs. R-CIRs, were pooled using a random-effects model. Heterogeneity was assessed using Chi-squared and I^2^ statistics.

**Results:**

Seventeen studies, mostly of moderate or high risk of bias (five in children, 10 in immunocompromised people, and two in those recently arrived) were included. In children, while in two studies, there was no significant difference between QFT-GIT and TST (≥5 mm) (pooled R-CIR = 1.11, 95% CI: 0.71, 1.74), two other studies showed QFT-GIT to outperform TST (≥10 mm) in identifying LTBI. In immunocompromised people, IGRA (T-SPOT.TB) was not significant different from TST (≥10 mm) for identifying LTBI, (pooled R-CIR = 1.01, 95% CI: 0.65, 1.58). The forest plot of two studies in recently arrived people from countries with high TB burden demonstrated inconsistent findings (high heterogeneity; I^2^ = 92%).

**Conclusions:**

Prospective studies comparing IGRA testing against TST on the progression from LTBI to TB were sparse, and these results should be interpreted with caution due to uncertainty, risk of bias, and unexplained heterogeneity. Population-based studies with adequate sample size and follow-up are required to adequately compare the performance of IGRA with TST in people at high risk of TB.

**Electronic supplementary material:**

The online version of this article (doi:10.1186/s12879-017-2301-4) contains supplementary material, which is available to authorized users.

## Background

The timely and accurate identification and prophylactic treatment of people with latent tuberculosis infection (LTBI) are important for controlling *Mycobacterium tuberculosis* (TB) worldwide. Once infected with LTBI, most people remain asymptomatic and are not contagious. However, 5-10% of those infected may progress to active TB in their lifetime and become infectious [1]. The risk of progression is higher in younger children [2], people who are immunocompromised or immunosuppressed [3, 4], and in people from countries with a high incidence of TB (≥40 cases per 100,000) [5].

There is no gold standard for the diagnosis of LTBI. Available screening tests provide indirect information on the presence of LTBI. Historically, the diagnosis of LTBI has relied on the use of the tuberculin skin test (TST) [6]. Recently, interferon gamma release assays (IGRAs) have been developed. These may overcome some of the limitations of TST (e.g., cross-reactivity in Bacilli Calmette-Guerin vaccinated people, error in measuring the size of induration of the skin reaction) and can be used as a replacement or adjunct to the TST. Currently, two IGRAs are commercially available: QuantiFERON-TB Gold In-tube (QFT-GIT) (Cellestis Ltd., Carnegie, Australia) and T-SPOT.TB (Oxford Immunotec Ltd, Oxford, UK).

Since the introduction of IGRAs, an increasing number of studies has compared their performance with TST for identification of LTBI. In the absence of a gold standard, these studies have measured a) the association between test results and surrogate measures (e.g., duration or proximity of exposure to an index TB case), b) compared specificity of tests in people at low risk of TB (e.g., healthy people or people from low TB incidence countries) or c) compared sensitivity of tests against culture-confirmed individuals with TB [6]. The results from these studies may be biased due to exposure misclassification. Moreover, the findings from studies using the diagnosis of TB as a marker for LTBI may also be biased, given the difference between the two entities.

Other studies have compared the strength of association between IGRA and TST test results in relation to the risk of progression to active TB. The comparison is based on the assumption that people with LTBI are at greater risk of progression to active TB compared to those without it. With this proxy measure, IGRA and TST tests have been compared for their ability to predict progression from LTBI to active TB. For example, two meta-analyses [1, 7] synthesised evidence from primary studies comparing IGRAs to TST using progression to active TB as a proxy for LTBI. Although this approach provides a potentially unbiased estimate of performance, these meta-analyses had methodological limitations. For example, the first meta-analysis included and synthesised studies in which IGRA or TST test positive people were treated with anti-TB prophylactic agents [7]. However evidence suggests that the currently available treatments for LTBI are effective in preventing a reactivation of TB (60–90%) [5], hence treatments would have had an independent impact on the performance of the IGRA and TST tests. In the second meta-analysis studies of ‘in-house’ assays were included [1]. Little is known about the quality and consistency of these tests across clinical laboratories (UK Standards for Microbiology Investigations) since they are not subject to the regulations of commercially developed tests. Finally, none of the two meta-analyses compared individual IGRAs to TST in predicting risk of progression to active TB separately in children, immunocompromised people, and those who have recently arrived from high TB burden countries.

In this systematic review we aimed to identify, appraise, and synthesise the relevant evidence from longitudinal cohort studies comparing performance of both types of IGRA to TST in identifying LTBI through predicting progression to active TB separately for children, immunocompromised people, and those who have recently arrived from high TB burden countries.

## Methods

This review was conducted as part of a clinical guideline commissioned by the National Institute for Health Research (NIHR) Health Technology Assessment (HTA) Programme (project number 13/178/01) [8].

### Inclusion and exclusion criteria

We included English language reports of head-to-head comparative cohort studies aimed at identifying LTBI which followed-up people to incidence of active TB after testing with IGRAs (QFT-GIT, T-SPOT.TB) and TST separately in children (<18 years), immunocompromised people (e.g., people with HIV, transplant recipients, people receiving or about to start anti-tumour necrosis factor TNF-α treatment), and people arriving from high incidence TB areas (annual incidence ≥ 40 per 100,000) [5]. We excluded studies of people treated with anti-tuberculosis prophylaxis after testing for LTBI, studies which used ‘in-house’ assays, and single-arm studies testing people for LTBI with only IGRAs or TST.

### Outcomes of interest

The proportion of people progressing to active TB.

### Search strategy

We searched MEDLINE (Ovid), The Cochrane Library, MEDLINE In-Process Citations and Daily Update (Ovid), EMBASE (Ovid), and Science Citation Index (Web of Knowledge). Searches were limited to English Language studies published between January 2009 and June 2015. Electronic searches were supplemented by manually searching reference lists of potentially relevant studies, contacting experts in the field and screening of manufacturers’ and other relevant websites. For unpublished studies, we searched specific conference proceedings for the last 5 years. Details of the search strategy can be found in Additional file 1.

### Study selection, data extraction, and risk of bias assessment

Two independent reviewers (AT and PA) screened the titles and abstracts of all identified articles, and afterwards full-texts of potentially relevant articles using pre-piloted forms. One reviewer (PA) extracted relevant data from included studies using a pre-piloted data extraction form. Data extraction was cross-checked by an independent reviewer (AT). Data were collected on author, year, country, and duration of follow-up, population characteristics (age, sex, sub-group), intervention (types of IGRAs), comparator (TST, cut-off values), Bacillus Calmette–Guérin (BCG) status, TB diagnosis, and outcomes (the proportion of people who progressed to active TB). Risk of bias was assessed using the Quality in Prognosis Studies (QUIPS) tool, developed to appraise studies reporting the associations between prognostic factors and health outcomes [9]. The tool addresses the risk of bias for six domains: patient selection/participation, study sample attrition, index test measurement, outcome/construct validity measurement, confounding, and statistical analysis/outcome reporting. Any disagreements at study selection, data extraction, and risk of bias assessment phases of the review were resolved by discussions between the two reviewers or through adjudication of a third independent reviewer.

### Data synthesis and analysis

Given the absence of a gold standard for diagnosing LTBI, the performance of tests was compared using alternative methodology which relies on the validation of test results against a predetermined validity construct (i.e. a proxy for a reference standard) – progression to active TB. For each test (IGRA or TST), the strength of association between test results and incidence of active TB was expressed using cumulative incidence ratios (CIRs; the ratio of active TB incidence in test positives versus TB incidence in test negatives) with corresponding 95% CIs. A statistically significant estimate of CIR > 1 would indicate that a test (IGRA or TST) has discriminatory power in predicting occurrence of active TB (i.e. of identifying LTBI). The effect measures comparing IGRAs to TST were summarised as ratios of CIRs (R-CIRs) for IGRA vs. TST with 95% CIs. A statistically significant estimate of R-CIR > 1 would suggest for example that an IGRA has a better power of predicting the occurrence of active TB (i.e., of identifying LTBI) than TST. Synthesised data were stratified by type of IGRA (QFT-GIT or T-SPOT.TB) and TST threshold (≥5 mm, ≥ 10 mm, ≥ 15 mm). We have not synthesised data from studies of QFT-G because this test is no longer commercially available. We used a random-effects model to pool the summary effect measure (R-CIR) across studies when deemed appropriate and feasible (e.g., no evidence of clinical and methodological heterogeneity, the same cut-off value of TST). We did not pool study results if there was evidence of important clinical or statistical heterogeneity or if data were insufficient. The presence of heterogeneity was judged by visual inspection of forest plots of R-CIRs (and degree of overlap across 95% CIs), formal statistical tests (Chi-square <0.10 and the I^2^ statistic >50%), or if data permitted a subgroup analysis with respect to a priori defined factors including: BCG vaccination status, risk of bias, TST threshold (≥5 mm, ≥ 10 mm, ≥ 15 mm) and prevalence of TB in country of origin. Publication bias exploration, where data permitted, was planned using asymmetry of contour-enhanced funnel plots from the meta-analyses [10].

## Results

### Study identification process

**Fig. 1:**
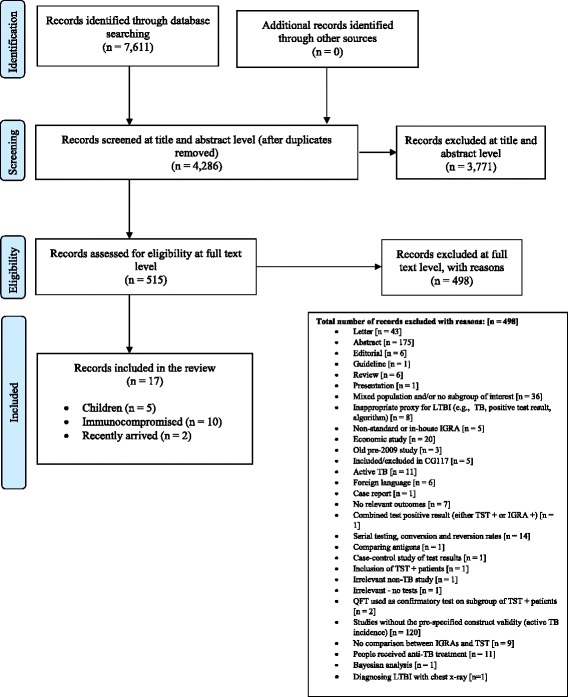
PRISMA [28] flow diagram

Of the 7,611 records identified, 515 were selected for full-text examination. Of these, 498 records were excluded. The remaining 17 publications were included in the review [11–27]. Figure 1 shows the study flow with reasons for exclusion depicted in the PRISMA flow diagram [28].

### Characteristics of included studies

Of the 17 included studies, five were conducted in children [11, 13–16], 10 in immunocompromised people [12, 17–25], and two studies [26, 27] were undertaken in people recently arrived from countries with a high incidence of TB. Most were prospective cohort studies although two [12, 18] (both in immunocompromised people) were retrospective cohorts. Further details on baseline characteristics of included studies are provided in Table 1.

**Table 1:** Characteristics of studies in children, immunocompromised and recently arrived immigrants from countries with high incidence of TB

Study ID (First author, year, country, and extent of TB burden^b^)	Tests compared	Total number of participants tested with IGRA and TST	Mean (range or SD) or median age (IQR) in years	BCG vaccination [n, (%)] in population	Mean or median length of follow-up (years)	Method(s) for diagnosing TB
Children						
Diel 2011 [11], Germany Low incidence	QFT-GIT vs. TST (5/10 mm)	126	Mean: 10.4 (SD: 4.3)	45 (35.7%)	2–4	Chest x-ray, identification of AFB in sputum samples by bronchoscopy or lavage of gastric secretions, conventional culture of Mycobacterium tuberculosis, nucleic acid amplification and/or histopathology, assessment of preceding clinical suspicion of TB
Mahomed 2011 [13], South Africa High incidence	QFT-GIT vs. TST (5 mm)	5244	NR (range: 12–18)	Yes: 4917 (93.8%); Unknown 281 (5.4%)	3.8	Two sputum samples for smear microscopy on two separate occasions. If any single sputum was smear positive, a mycobacterial culture, chest x-ray, and HIV test were performed
Metin Timur 2014 [14], Turkey Intermediate incidence	QFT-GIT vs. TST (15 mm)	81	Mean: 7.9 (range: 0.5–16)	69 (85.2%)	3	TST and QFT-GIT test positive in a child who had symptoms of TB disease and/or abnormal findings on chest radiograph, CT or proven M. tuberculosis culture, PCR or histo- pathological examination
Noorbakhsh 2011 [15], Iran Intermediate incidence	QFT-G vs. TST (10 mm)	Not reported	NR (<20)	Not reported	1	Person diagnosed by an internist in the pulmonary and infectious ward of hospital.
Song 2014 [16], South Korea High incidence	QFT-GIT vs. TST (10/15 mm)	2982	Mean: 15.1 (SD: 1.3)	1,818 (61.0%)	2	NR
Immunocompromised						
Elzi 2011 [12], Switzerland (PLWHIV) Low incidence	T-SPOT.TB vs. TST (5 mm)	64	Median: 33 (IQR: 31–42)	NR	2	NR
Kim 2011 [17], South Korea (Post kidney transplantation) High incidence	T-SPOT.TB vs. TST (5 mm)	272	NR (range: 40.4–46.0)	215 (79%)	1.17 (median)	Symptoms/signs, sputum AFB smear, and a CT scan
Kim 2015 [18], South Korea (Rheumatoid diseases)^a^ High incidence	QFT-GIT vs. TST (5 mm)	282	Mean: 46.0 (SD: 15.4)	NR	4	Medical records of clinical features, sputum or tissue acid-fast bacilli staining and radiological findings
Lee 2009 [19], Taiwan (Haemodialysis in end-stage renal disease (ESRD)) High incidence	QFT-G vs. TST (10 mm) T-SPOT.TB vs. TST (10 mm)	32	Mean: 53.8 (range: 34.4–77.7)	53 (82.8%)	2	Sputum TB smear, culture and chest radiography
Lee 2014 [22], South Korea (Haematopoietic stem cell transplantation recipients) High incidence	QFT-GIT vs. TST (10/15 mm)	169	Mean: 42.3 (SD: 13.8)	353 (90.7%)	1.3 (median)	Chest x-ray, a sputum AFB smear and CT scan (pulmonary TB)
Lee 2015 [23], South Korea (People with inflammatory arthritis) High incidence	QFT-GIT vs. TST (10 mm)	342	Median: 40 (IQR: 30–53)	236 (69.0%)	3.5 (median)	Pulmonary TB was confirmed by sputum or bronchial washing culture
Milman 2011 [20], Denmark (People with sarcoidosis) Low incidence	QFT-G vs. TST (10 mm)	41	Median: 39 (IQR: 25–39)	12 (27.3%)	5	Examinations of tissue specimens, Culture confirmed and polymerase chain reaction
Moon 2013 [21], South Korea (Haematopoietic stem cell transplantation candidates) High incidence	QFT-GIT vs. TST (5 mm)	244	Mean: 47 (range: 35–55)	201 (82%)	0.8 (median)	NR
Sester 2014 [24], Various European healthcare facilities (PLWHIV, chronic renal failure, rheumatoid arthritis, solid-organ transplant or stem-cell transplantation) Low incidence	QFT-GIT vs. TST (5 mm) T-SPOT.TB vs. TST (5 mm)	1282	NR	NR	5	Signs and symptoms of active TB. Culture confirmed and polymerase chain reaction
Sherkat 2014 [25], Iran (Haemodialysis in end-stage renal disease (ESRD)) Intermediate incidence	T-SPOT.TB vs. TST (10 mm)	Not reported	Mean: 44 (SD: 15.5)	12 (27.3%)	1.75	NR
Recent arrivals from countries with a high incidence of TB						
Harstad 2010 [26], Norway Low incidence	QFT-GIT vs. TST (6/15 mm)	Not reported	NR	NR	2.67	NR
Kik 2010 [27], The Netherlands Low incidence	QFT-GIT vs. TST (10/15 mm) T-SPOT.TB vs. TST (10/15 mm)	339	NR	274 (80.8%)	2	Chest radiography, symptoms, smear and/or culture results

*NR* not reported, *AFB* acid-fast bacilli, *BCG* bacille calmette-guérin, *CT* computed tomography, *IQR* interquartile range, *N*/*A* not applicable, *PCR* polymerase chain reaction, *PLWHIV* people living with human immunodeficiency virus, *QFT*-*G* quantiferon gold, *QFT*-*GIT* quantiferon gold-in-tube, *SD* standard deviation, *TB* tuberculosis, *TST* tuberculin skin test

^a^One unique study but three sub-groups received testing (TST alone, QFT-GIT alone and TST and QFT-GIT simultaneously)

^b^Low incidence of TB- ≤ 20 cases per 100,000; intermediate incidence of TB- > 20 cases per 100,000 < 40 cases per 100,000; high incidence of TB- ≥40 cases per 100,000

#### Children

The five studies were undertaken in Germany [11], Turkey [14], Iran [15], South Africa [13] and South Korea [16].

Three studies [11, 13, 16] compared QFT-GIT with TST (5 mm/10 mm). One study [15] compared QFT-G with TST (10 mm). The prevalence of BCG vaccination was reported in three studies as ranging from 36 to 94% [11, 13, 16]. The mean length of follow-up to diagnosis of active TB ranged from 1 year [15] to 4 years [11, 13]. Three studies [11, 13, 15] clearly stated the method(s) used to diagnose TB.

#### Immunocompromised people

Six of the 10 studies were conducted in South Korea and Taiwan [17–19, 21–23], one each in Iran [25], Switzerland [12], and Denmark [20] and the remaining study across various European countries [24].

In two studies, participants were receiving haemodialysis for end-stage renal disease (ESRD) [19, 25]. Two other studies included haematopoietic stem cell transplantation candidates [21] and haematopoietic stem cell transplantation recipients [22]. The remaining six studies included people with ‘rheumatic disease’ [18], people who had undergone kidney transplantation [17], people living with human immunodeficiency virus (PLWHIV) [12], people being treated for inflammatory arthritis [23], people being treated for sarcoidosis [20], and participants with various conditions and diseases (PLWHIV, chronic renal failure, rheumatoid arthritis, solid-organ transplant or stem-cell transplantation) [24].

Four studies compared T-SPOT.TB to TST (5 mm/10 mm) [12, 17, 19, 25], two studies QFT-G to TST (10 mm) [19] or TST (6 mm/12 mm) [20], four studies compared QFT-GIT to either TST (5 mm) [18, 21] or TST 10 mm/15 mm [22, 23]. The study undertaken by Sester and colleagues [24] compared three tests (TST measured at 5 mm, QFT-GIT and T-SPOT.TB). The mean follow-up duration across studies ranged from 1.2 to 5 years. Seven studies [17–20, 22–24] reported methods for TB diagnosis.

#### People who recently arrived from countries with high TB incidence

We identified only two studies [26, 27] conducted in people recently arriving from high TB incidence countries. These studies were undertaken in Norway [26] and the Netherlands [27]. The Harstad et al. study [26] included adult asylum seekers and the Kik et al. [27] study adults who were recently exposed to infectious pulmonary TB. Most of the participants in both studies had arrived from Europe, Africa, and Asia. The studies compared QFT-GIT with TST (≥6 mm and ≥15 mm) [26] and QFT-GIT/T-SPOT.TB with TST (≥10 mm and ≥ 15 mm) [27]. The prevalence of BCG vaccination was reported in only one of the studies at 81% [27]. Mean length of follow-up ranged from 2 years [27] to 3 years [26]. Only one study provided sufficient information on method(s) used to diagnose TB, which included chest radiography, symptoms, smear and/or culture results [27].

### Assessment of risk of bias

The risk of bias by domain and overall is presented in Table 2. In children, two studies [14, 15] had a high risk and the remaining three studies a moderate risk of bias [11, 13, 16]. Most studies had a moderate risk of bias for misclassification of individuals in relation to construct validity groups, since no clear definitions and ascertainment methods were provided [11, 13, 15]. In immunocompromised people, three studies had an overall high [12, 19, 25] and another three a moderate risk of bias [21, 22, 24]. The remaining four studies had a low overall risk of bias [17, 18, 20, 23]. Five studies [12, 19, 21, 22, 25] had moderate/high risk of bias for the items of study participation, outcome measurement and study confounding.

**Table 2:** Risk of bias in studies of active TB incidence comparing IGRA with TST in children, immunocompromised people and recently arrived people from countries with a high incidence of TB

First author, Year	Study Participation	Study Attrition	Prognostic Factor Measurement	Outcome/Construct Measurement	Study Confounding	Statistical Analysis and Reporting	Total ROB
Children							
Diel, 2011 [11]	Low	Low	Moderate	Moderate	Low	Low	Moderate ROB
Mahomed, 2011 [13]	Moderate	Moderate	Moderate	Moderate	High	Low	Moderate ROB
Metin Timur 2014 [14]	High	High	Moderate	Moderate	High	High	High ROB
Noorbakhsh 2011 [15]	High	High	High	Moderate	High	High	High ROB
Song, 2014 [16]	Low	Moderate	Low	High	Moderate	Low	Moderate ROB
Immunocompromised							
Elzi, 2011 [12]	High	Low	Low	Moderate	High	Low	High ROB
Kim, 2011 [17]	Low	Low	Low	Low	Moderate	Low	Low ROB
Kim, 2015 [18]	Low	Low	Low	Low	High	Low	Low ROB
Lee, 2009 [19]	High	Low	Low	Moderate	High	Low	High ROB
Lee, 2014 [22]	High	Moderate	Moderate	Moderate	Low	Low	Moderate ROB
Lee, 2015 [23]	Low	Low	Moderate	Low	High	Low	Low ROB
Milman, 2014 [20]	Low	Low	Moderate	Low	Low	Low	Low ROB
Moon, 2013 [21]	Moderate	Low	Moderate	Moderate	Moderate	Low	Moderate ROB
Sester, 2014 [24]	Low	Low	Moderate	Low	High	Low	Moderate ROB
Sherkat, 2014 [25]	High	High	Moderate	High	High	Moderate	High ROB
Recent arrivals from countries with a high incidence of TB							
Harstad, 2010 [26]	High	Low	High	Moderate	High	High	High ROB
Kik, 2010 [27]	Low	Low	Low	Low	Low	Low	Low ROB

*IGRA* interferon gamma release assay, *ROB* risk of bias, *TST* tuberculin skin test

Risk of bias item responses (per domain, overall): high, moderate or low

Of the two studies in a recently arrived people from high TB burden countries, one study had a high overall risk of bias [26] and the other, low risk of bias [27]. In the Harstad study [26], high risk of bias was noted in most of the bias domains (e.g., the study participation, prognostic factor measurement, study confounding, and statistical analysis and reporting domains).

### The incidence of active TB following the testing for LTBI by subgroups of interest

Details on incidence of active TB by LTBI test results are presented for the subgroups of interest in Table 3. IGRAs and TST (5 mm) were both significantly effective across studies in detecting LTBI for children and immunocompromised people. Among immunocompromised people and those recently arrived from high incidence countries findings were not statistically significant for TST (10 mm) in predicting progression to active TB. Among recent arrivals, T-SPOT.TB test results were also not statistically significantly associated with progression to active TB.

**Table 3:** Progression to TB following LTBI testing with IGRAs and TST in children, immunocompromised and recently arrived immigrants

Study ID (First author, year)	Total test results available	Type of IGRA test and TST (thresholds)	Number of people with positive results	Number of people with negative results	People with test positive results who progressed to TB (n)	People with test negative results who progressed to TB (n)
Children						
Diel, 2011 [11]	104	QFT-GIT	21	83	6	0
		TST (≥5 mm)	40	64	6	0
		TST (≥10 mm)	40	64	4	2
Mahomed, 2011 [13]	5244	QFT-GIT	2669	2575	39	13
		TST (≥5 mm)	2894	2350	40	12
Metin Timur, 2014 [14]	69	QFT-GIT	0	69	0	0
		TST (≥15 mm)	69	0	0	0
Noorbakhsh, 2011 [15]	59	QFT-G	18	41	10	0
	58	TST (≥10 mm)	8	50	3	7
Song, 2014 [16]	2966	QFT-GIT	317	2649	11	12
	2982	TST (≥10 mm)	663	2319	13	10
		TST (≥15 mm)	231	2751	13	10
Immunocompromised						
Elzi, 2011 [12]	43	T-SPOT.TB	25	18	25	18
	44	TST (≥5 mm)	22	22	22	22
Kim, 2011 [17]	265	T-SPOT.TB	89	176	4	0
	288	TST (≥5 mm)	26	262	1	3
Kim, 2015^a^ [18]	282	QFT-GIT	7	275	0	1
	282	TST (≥5 mm)	12	270	0	1
Lee, 2009 [19]	30	QFT-G	12	18	1	0
		T-SPOT.TB	15	17	0	2
		TST (≥10 mm)	20	12	1	1
Lee, 2014 [22]	159	QFT-GIT	26	133	3	2
	169	TST (≥10 mm)	19	150	0	5
		TST (≥15 mm)	12	157	0	5
Lee, 2015^b^ [23]	342	QFT-GIT	103	239	N/A	4
	239	TST (≥10 mm)	60	179	2	2
Milman, 2011 [20]	41	QFT-G	0	41	0	0
	12	TST (≥10 mm)	0	12	0	0
Moon, 2013 [21]	210	QFT-GIT	40	170	1	1
	244	TST (≥5 mm)	39	205	0	2
Sester, 2014 [24]	1238	QFT-GIT	159	1079	3	5
	1217	T-SPOT.TB	193	1024	4	6
	1282	TST (≥5 mm)	149	1133	4	7
Sherkat, 2014 [25]	44	T-SPOT.TB	6	38	1	0
		TST (≥10 mm)	8	36	1	0
Recent arrivals from countries with a high incidence of TB						
Harstad, 2010 [26]	815	QFT-GIT	238	577	8	1
		TST (≥6 mm)	415	395	8	1
	813	TST (≥15 mm)	121	692	3	6
Kik, 2010 [27]	327	QFT-GIT	178	149	5	3
	299	T-SPOT.TB	181	118	6	2
	339	TST (≥10 mm)	288	51	9	0
	322	TST (≥15 mm)	184	138	7	1

*N*/*A* not applicable, *QFT*-*G* quantiferon gold, *QFT*-*GIT* quantiferon gold-in-tube, *TB* tuberculosis, *TST* tuberculin skin test, *n* number

^a^One unique study but three sub-groups received testing (TST alone, QFT-GIT alone and TST and QFT-GIT simultaneously)

^b^People with a positive result on QFT-GIT received TB preventative treatment

### Children

#### QFT-GIT

Fifty-six of the 3007 (1.86%) QFT-GIT-positive children (4 studies [11, 13, 14, 16]) progressed to active TB compared with 25 of the 5376 (0.46%) QFT-GIT-negative children (overall crude CIR for QFT-GIT: 1.86/0.46 = 4.01, 95% CI: 2.51, 6.40).

#### TST (5 mm)

Forty-six of the 2934 (1.56%) TST (≥5 mm)-positive children (2 studies [11, 13]) progressed to TB compared with 12 of 2414 (0.49%) TST (<5 mm)-negative children of whom only 12 (0.49%) progressed to active TB (overall crude CIR for TST-5 mm: 1.57/0.50 = 3.14, 95% CI: 1.68, 5.94).

#### TST (10 mm)

Twenty of the 711 (2.81%) TST (≥10 mm)-positive children (3 studies [11, 15, 16]) progressed to TB compared with 19 of the 2433 (0.78%) TST (<10 mm)-negative children (crude CIR for TST-10 mm: 2.81/0.78 = 3.60, 95% CI: 1.93, 6.71).

### Immunocompromised

#### IGRAs (QFT-GIT and T-SPOT.TB)

In the immunocompromised population (4 studies [18, 21, 22, 24]), seven of the 232 (3.02%) QFT-GIT positive people progressed to active TB compared with 13 of the 1999 (0.65%) QFT-GIT that tested negative (crude CIR for QFT-GIT: 3.02/0.65 = 4.65, 95% CI: 1.87, 11.51). 34 of the 328 (10.37%) T-SPOT.TB positive people (5 studies [12, 17, 19, 24, 25]) progressed to TB compared with 26 of the 1273 (2.04%) T-SPOT.TB that tested negative (crude CIR for T-SPOT.TB: 10.37/2.04 = 5.08, 95% CI: 3.09, 8.33).

#### TST (10 mm)

Four of the 107 (3.74%) people with TST (≥10 mm) (5 studies [19, 20, 22, 23, 25]) progressed to TB compared with eight of 389 (2.06%) with TST (<10 mm) (crude CIR for TST-10 mm: 3.74/2.06 = 1.82, 95% CI: 0.58, 5.92).

### Recent arrivals from countries with a high incidence of TB

#### IGRAs (QFT-GIT and T-SPOT.TB)

Across two studies [26, 27], 13 of 416 (3.13%) recent arrivals who tested positive with QFT-GIT progressed to TB compared to four of 726 (0.55%) that tested negative (crude CIR for QFT-GIT: 3.13/0.55 = 5.69, 95% CI: 1.86, 17.28). Six of 181 (3.31%) people who tested positive with T-SPOT.TB progressed to TB compared to two of 118 (1.69%) that tested negative (crude CIR for T-SPOT.TB: 3.31/1.69 = 1.96, 95% CI: 0.40, 9.53).

#### TST (≥6 mm or ≥10 mm)

In one study [26] TST (≥6 mm) was used as the threshold for a positive test. Results showed that eight of 415 (1.93%) people who tested positive progressed to TB compared with one of the 395 (0.25%) people who tested negative (crude CIR for TST-6 mm: 1.93/0.25 = 7.72, 95% CI: 0.96, 60.59). In the other study [27], 9 of the 288 (3.12%) people with a TST (≥10 mm) progressed to TB as compared to none of the 51(0%) people that tested negative (crude CIR for TST-10 mm: 3.42, 95% CI: 0.20, 57.83).

### Comparative performance of tests for identifying latent tuberculosis infection

#### Children

##### QFT-GIT vs. TST (≥5 mm)

**Fig. 2:**
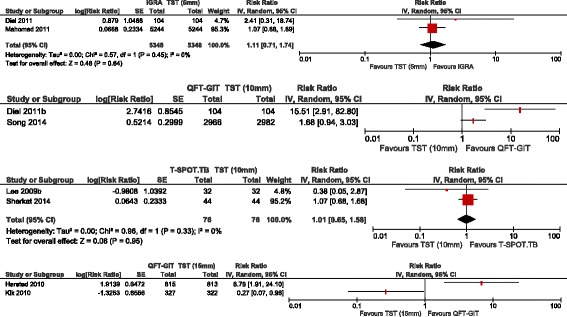
Pooled cumulative incidence ratios for IGRAs versus TST in children, immunocompromised and recently arrivals. **a** Pooled cumulative incidence ratio of QFT-GIT versus TST (5 mm) for a child population. **b** Forest plot of cumulative incidence ratio of QFT-GIT versus TST (10 mm) for a child population. **c** Pooled cumulative incidence ratio of T-SPOT.TB versus TST (10 mm) for an immunocompromised population. **d** Forest plot of cumulative incidence ratio of QFT-GIT versus TST (15 mm) for a recent arrival population

Only two studies were eligible for pooling R-CIRs and 95% CIs to compare QFT-GIT and TST (≥5 mm) [11, 13]. The meta-analytic estimate was not statistically significant between QFT-GIT and TST (≥5 mm) for identifying LTBI (Fig. 2a; pooled CIR = 1.11, 95% CI: 0.71, 1.74).

##### QFT-GIT vs. TST (≥10 mm)

The individual results from two studies tended to favour IGRA (QFT-GIT) to TST (≥10 mm) [11, 16] (Fig. 2b). We did not pool the R-CIRs due to significant heterogeneity across the estimates of these studies (*p* = 0.01, I^2^ = 83%). Both studies were at moderate risk of bias, therefore the risk of bias is less likely to explain this heterogeneity. One potential source of heterogeneity may have been the difference in the burden of TB incidence between the two studies. Specifically, the study which showed non-significant difference between IGRA and TST [16] was conducted in a high incidence area (South Korea) as opposed to the study by Diel et al. (2011) [11], which was conducted in low TB incidence area (Germany). There has been evidence showing reduced sensitivity and specificity of IGRAs in high compared to low TB burden areas, where the former is represented by high BCG vaccination rates given at birth [29–33].

### Immunocompromised people

#### T-SPOT.TB vs. TST (≥10 mm)

The R-CIRs were pooled across two studies that included an ESRD population (Fig. 2c; pooled R-CIR = 1.01, 95% CI: 0.65, 1.58) [19, 25]. The meta-analytic estimate comparing the performance between IGRA (T-SPOT.TB) and TST (≥10 mm) was not statistically significant. The corresponding R-CIRs for individual studies were also non-significant: 0.38 (95% CI: 0.05, 2.87) [19] and 1.07 (95% CI: 0.68, 1.68) [25]. We did not pool the study estimates across different immunocompromised populations due to clinical heterogeneity.

### People who arrived recently from countries of high TB burden

#### QFT-GIT vs. TST (≥15 mm)

Two studies compared QFT-GIT to TST (≥15 mm) for this population [26, 27]. As Fig. 2d suggests, in the Harstad et al. study [26], QFT-GIT was in favour over TST (R-CIR = 6.78, 95% CI: 1.91, 24.10). In contrast, in the Kik et al. study [27], TST was in favour over QFT-GIT (R-CIR = 0.27, 95% CI: 0.07, 0.96). The R-CIR estimates were not pooled due to significant heterogeneity arising from the opposing findings (Fig. 2d; *p* <0.01, I^2^ = 92%). The a priori defined factors (BCG vaccination status, TST threshold, risk of bias, and prevalence of TB in country of origin) could not readily explain this inconsistency. Note that the two studies differed in the study populations of asylum seekers [26] vs. immigrants with known contact with an index case [27]. Moreover, the Kik study [27] excluded contacts with TST <5 mm which may have influenced test accuracy parameter estimates.

## Discussion

This systematic review compared the performance of IGRAs with TST for identifying LTBI in terms of predicting progression to active TB in children, immunocompromised people and people who had recently arrived from high TB burden countries. There was limited evidence; mostly from studies with moderate to high risk of bias making it difficult to draw definitive conclusions. There was largely consistent evidence in favour of each test predicting progression to active TB, but there was evidence demonstrating that one test outperformed others. Even within the well-defined population categories of this study, there was a great deal of heterogeneity across the R-CIR effect estimates comparing IGRAs to TST, thereby rendering results inconclusive.

There was no evidence indicating that QFT-GIT was better or worse than TST (5 mm) in detecting LTBI in children. This should not be interpreted as the absence of difference, since the 95% CIs were wide enough to cover differences of at least moderate size in each direction either favouring IGRA or TST. When QFT-GIT was compared with TST (10 mm), the individual study estimates tended to favour QFT-GIT over TST, but there was still strong heterogeneity across the studies. One study [16] showed a non-significant difference between QFT-GIT and TST (10 mm) in a high TB burden setting and the other [11] favoured QFT-GIT over TST (10 mm) in a low TB burden setting. This observation is consistent with a growing body of evidence showing a reduced sensitivity and specificity of IGRAs in high compared with low TB burden areas, the former represented mostly by developing countries [29–33]. This heterogeneity in test performance might be explained by a number of factors relevant to these high TB burden settings for example BCG vaccination is frequently given at birth or there may be a higher frequency of exposure to MTB, different TB transmission dynamics, malnutrition, comorbidity, co-infection with HIV, exposure to non-tuberculous mycobacterium (NTMs) or helminthic infection [32–34].

Similarly, there was no evidence indicating that T-SPOT.TB was better or worse than TST (10 mm) in detecting LTBI in immunocompromised people. Again, 95% CIs were compatible with a wide range of values of moderate size in both directions.

The findings in two meta-analysed studies of recently arrived populations from high TB burden areas were in opposite direction. Specifically, one study [27] demonstrated that TST (15 mm) outperformed QFT-GIT, while the other study [26] showed the opposite. The a priori defined factors (TST threshold, BCG vaccination, risk of bias and TB burden) could not readily explain the inconsistency between these study findings. Other factors, such as inclusion criteria for study population could have contributed to this difference. For example, one study included asylum seekers [26] as opposed to the other study which included immigrants who had contacts with an index case [27]. In addition, the Kik study excluded contacts with TST <5 mm [27].

Despite the extensive research in this area, limited evidence is available on progression to TB in untreated populations following testing with commercial IGRAs/TST. This is likely to be a reflection of the standard of care in high-income countries which is to offer anti-tuberculous treatment to people who test positive. Moreover, some evidence has indicated that there exists variability in TB diagnosis across countries and studies, which further complicates the comparison of diagnostic accuracy of TB detection tests [35, 36].

The main strength of this systematic review is that it synthesises the available evidence on progression to TB in people who have not received anti-TB treatment for LTBI. Moreover, in this review we have evaluated and compared the performance of IGRA and TST tests separately in subgroups of children, immunocompromised, or recently arrived people from countries with high TB burden.

We identified two [1, 7] systematic reviews and meta-analyses assessing IGRAs for predicting the incidence of TB. The first review [1] included studies which used ‘in-house’ assays to diagnose LTBI, but little is known about the quality and consistency of these tests across the clinical laboratories (UK Standards for Microbiology Investigations). In addition, people who had indeterminate results at baseline and progressed to TB were assumed to have a negative result. Using this method would decrease the sensitivity and increase the specificity of the test. The second review [7] included studies where people received anti-TB preventative treatment. These studies may be biased as this therapy will decrease the number of people progressing to TB, underestimating the magnitude of the effect estimate for any given test. Since progression to TB is being used as a reference standard, this will have an impact on the predictive values or sensitivity and specificity of the test. In our review we included only studies in which people were not treated with anti-TB prophylactic treatment and were followed-up to identify progression of TB.

This review has limitations. First, we excluded studies on incidence of TB following serial testing with IGRAs/TST. If some time has passed since a person becomes infected with *M. bacterium* they may have a negative TST result on initial testing. However, on subsequent TST an individual may have a positive reaction because the initial test stimulates their ability to react to the test. This is commonly referred to as the ‘booster phenomenon.’ Unlike TST, the use of IGRAs in serial testing does not lead to a ‘booster phenomenon.’ Despite this, studies using IGRAs to assess reproducibility can potentially lead to conversion/reversion of test results, and this can alter the clinical decision on whether people should be treated for LTBI. Second, included studies did not stratify results by BCG status, so we were unable to present information on people with or without BCG vaccination who tested on IGRA/TST and further developed TB. Likewise, due to sparse data in our meta-analyses (maximum of only two studies pooled), we were unable to construct funnel plots to investigate the effects of publication bias.

More large prospective longitudinal studies or trials comparing head-to-head IGRAs versus TST in untreated populations would help to elucidate the relative merits of IGRA and TST tests in identifying LTBI among different population subgroups. We are aware of one study, the UK Prognostic Evaluation of Diagnostic IGRA Consortium (PREDICT) [37], which will add to existing knowledge as soon as information becomes available. However, there may not be many others given the increasing likelihood of treatment for those testing positively.

Given our findings that tests work but that there is a lack of evidence on which works best, policy makers and those selecting tests should consider practical issues such as the patient population, the availability of tests, and the patient acceptability of the tests [38]. More specifically, the knowledge of sensitivity and specificity of each IGRA and TST for identifying LTBI would also be advantageous. Should decision-makers decide to test, sensitivity and specificity estimates would provide valuable information on the cost-effectiveness of strategies to identify LTBI which progresses to TB in these populations.

## Conclusions

Longitudinal studies exploring progression rates from LTBI to active TB in children, immunocompromised people, and those recently arrived from areas of high TB burden are sparse. The pooled risk ratios in our analyses did not allow identification of superiority or of non-inferiority of the different tests investigated. Our findings are based on a limited number of studies comparing IGRAs with TST, and the results should be interpreted with caution due to uncertainty, risk of bias, and heterogeneity. Prospective population-based studies or trials with an adequate sample size and follow-up should be conducted in people who are considered to be at high risk for TB. These studies should employ standard diagnostic methodology and criteria for ascertaining incident cases of active TB. However, there may be difficulties in conducting such studies due to the increasing use of treatment for those who test positive.

## Additional file

Additional file 1: An example of the search strategy used to identify relevant papers. (DOCX 16 kb)

## Abbreviations

BCG: Bacillus Calmette–Guérin
CI: Confidence intervals
CIR: Cumulative incidence ratio
ESRD: End-stage renal disease
HTA: Health technology assessment
IGRA: Interferon gamma release assay
LTBI: Latent tuberculosis infection
NIHR: National institute for Health Research
NTM: Non-tuberculous mycobacterium
PICO: Population, intervention, comparator and outcome
PLWHIV: People living with human immunodeficiency virus
PRISMA: Preferred reporting items for systematic reviews and meta-analyses
QFT-G: QuantiFERON®-TB Gold
QFT-GIT: QuantiFERON®-TB Gold In-Tube test
QUIPS: Quality in prognosis studies
R-CIR: Ratio of cumulative incidence ratio
ROB: Risk of bias
TB: *Mycobacterium* Tuberculosis
TNF: Tumour necrosis factor
TST: Tuberculin skin test
