# Supplementary material for: Comparing interferon-gamma release assays with tuberculin skin test for identifying latent tuberculosis infection that progresses to active tuberculosis: systematic review and meta-analysis
Source: BMC Infect Dis. 2017 Mar 9;17:200. doi: 10.1186/s12879-017-2301-4 (PMC5343308; doi:10.1186/s12879-017-2301-4)
Supplement: Additional file 1: — An example of the search strategy used to identify relevant papers. (DOCX 16 kb) [file 12879_2017_2301_MOESM1_ESM.docx]

Appendix 1

Example of the search strategy undertaken.

**Medline**

Ovid MEDLINE(R) 1946 to March Week 2 2014, searched on 21/03/2014

| 1 | (laten* adj3 (tb* or tubercul*)).tw. | 2701 |
| --- | --- | --- |
| 2 | ltb*.tw. | 6939 |
| 3 | tubercul*.tw. | 158617 |
| 4 | Tuberculosis/ | 51049 |
| 5 | Latent Tuberculosis/ | 866 |
| 6 | Tuberculosis, Pulmonary/ | 63874 |
| 7 | Mycobacterium tuberculosis/ | 35401 |
| 8 | 1 or 2 or 3 or 4 or 5 or 6 or 7 | 195420 |
| 9 | quantiferon*.tw. | 819 |
| 10 | QFT*.tw. | 557 |
| 11 | t spot*.tw. | 261 |
| 12 | exp Enzyme-Linked Immunosorbent Assay/ | 122317 |
| 13 | Interferon-gamma Release Tests/ | 377 |
| 14 | ((interferon* or IFN*) adj3 gamma* adj3 (release* or test* or assay*)).tw. | 3856 |
| 15 | ((y-interferon or interferon-y) adj3 (release* or test* or assay*)).tw. | 7 |
| 16 | IGRA*.tw. | 448 |
| 17 | 9 or 10 or 11 or 12 or 13 or 14 or 15 or 16 | 126231 |
| 18 | 8 and 17 | 3837 |
| 19 | Latent Tuberculosis/di | 576 |
| 20 | 18 or 19 | 4058 |
| 21 | Animals/ not Humans/ | 3812070 |
| 22 | 20 not 21 | 3477 |
| 23 | limit 22 to english language | 3011 |
| 24 | limit 23 to ed=20091207-20140321 | 1285 |

Total after duplicates removed: 1218

Updated the Medline search from the date it was run to the date of the Medline In-Process search when auto-alerts were started.

Line 24 = limit 23 to ed=20140322-20140409: 3

Update search Dec 2014

Ovid MEDLINE(R) 1946 to November Week 3 2014, searched on 02/12/2014

Re-ran search above with the following limit:

Line 24 = limit 23 to ed=20140409-20141202: 222

Update search Jun 2015

Ovid MEDLINE(R) 1946 to June Week 3 2015, searched on 29/06/2015

Re-ran search above with the following limit:

Line 24 = limit 23 to ed=20141202-20150629: 194
